# Elicitor-Induced Biochemical and Molecular Manifestations to Improve Drought Tolerance in Rice (*Oryza sativa* L.) through Seed-Priming

**DOI:** 10.3389/fpls.2017.00934

**Published:** 2017-06-06

**Authors:** Mahesh K. Samota, Minnu Sasi, Monika Awana, Om P. Yadav, S. V. Amitha Mithra, Aruna Tyagi, Suresh Kumar, Archana Singh

**Affiliations:** ^1^Division of Biochemistry, ICAR-Indian Agricultural Research InstituteNew Delhi, India; ^2^ICAR-National Research Centre on Plant BiotechnologyNew Delhi, India

**Keywords:** drought responsive gene, drought stress, elicitor, seed-priming, transcription factor

## Abstract

Rice (*Oryza sativa* L.) is one of the major grain cereals of the Indian subcontinent which face water-deficit stress for their cultivation. Seed-priming has been reported to be a useful approach to complement stress responses in plants. In the present study, seed-priming with hormonal or chemical elicitor [*viz*. methyl jasmonate (MJ), salicylic acid (SA), paclobutrazol (PB)] showed significant increase in total phenolic content, antioxidant activity and expression of *R*ice *D*rought-responsive (*RD1* and *RD2*) genes (of AP2/ERF family) in contrasting rice genotypes (Nagina-22, drought-tolerant and Pusa Sugandh-5, drought-sensitive) under drought stress. However, decrease in lipid peroxidation and protein oxidation was observed not only under the stress but also under control condition in the plants raised from primed seeds. Expression analyses of *RD1* and *RD2* genes showed upregulated expression in the plants raised from primed seeds under drought stress. Moreover, the *RD2* gene and the drought-sensitive genotype showed better response than that of the *RD1* gene and the drought-tolerant genotype in combating the effects of drought stress. Among the elicitors, MJ was found to be the most effective for seed-priming, followed by PB and SA. Growth and development of the plants raised from primed seeds were found to be better under control and drought stress conditions compared to that of the plants raised from unprimed seeds under the stress. The present study suggests that seed-priming could be one of the useful approaches to be explored toward the development of simple, cost-effective and farmer-friendly technology to enhance rice yield in rainfed areas.

## Introduction

Rice (*Oryza sativa* L.) a drought-sensitive crop exhibits impeded growth and development when exposed to water-deficit stress at critical growth stages feeds more than three billion people and provides 50–80% of the daily calories intake (Khush, 2005). Plants respond to water stress via a series of biochemical, physiological and molecular processes. Evidences suggest that under multiple stresses plants exhibit biochemical and molecular responses in addition to the shared hormonal responses as part of their stress tolerance strategies (Ramegowda and Senthil-Kumar, 2015). In many Asian countries, including India rice is generally grown by direct seeding in rainfed areas because it reduces input costs, improves resource use efficiency and improves system productivity (Liu et al., 2015). However, poor germination and stand-establishment of direct-seeded rice under the unfavorable environmental conditions remains a major impediment.

Although development of drought-tolerant crop varieties has been a challenging task, progress has been made in this direction with identification of certain bottlenecks. Therefore, alternative strategies to improve drought tolerance in rice are required to meet the increasing demand of rice when uneven distribution of rainfall and extreme weather conditions are observed frequently due to the climate change. Seed-priming, a controlled hydration technique that induces pre-germination metabolism without allowing radicle emergence, has emerged as a practical and effective approach to enhance tolerance against various abiotic stresses (Jisha et al., 2013; Paparella et al., 2015). Chen et al. (2010) reported that seed-priming enables the seedling to cope with environmental stresses by vigorous head-start or/and cross tolerance. Seed-priming affects biochemical and physiological processes (synthesis of nucleic acids and proteins, DNA repair, antioxidant activity and energy metabolism) leading to higher stress tolerance ability in the seedlings (Varierf et al., 2010; Hasanuzzaman and Fujita, 2011; Hussain et al., 2016b). Seed-priming has been demonstrated to enhance abiotic stresses tolerance, including drought, salinity, chilling and heavy metals, in different plant species (Ashraf and Rauf, 2001; Farooq et al., 2008; Bayat and Sepehri, 2012; Khan et al., 2012; Hussain et al., 2016a). Seed-priming of wheat with salicylic acid (SA) increased moisture content, dry matter yield, antioxidant activity and total chlorophyll content under drought stress (Singh and Usha, 2003), and selenium (Se) priming was reported to enhance drought tolerance in rapeseed by regulating enzymatic and non-enzymatic antioxidant activities (Hasanuzzaman and Fujita, 2011). Recently, seed-priming with selenium (Se) and SA was reported to be more effective in protecting rice plants against chilling stress (Hussain et al., 2016b). Thus, seed-priming with plant hormone and chemical compound is found to be effective in assuaging the damaging effects of abiotic stress in plants. Moreover, the biochemical, physiological and molecular basis of seed-priming to mitigate abiotic stresses in plants is becoming clear day-by-day.

Among the classical plant hormones (see Kumar et al., 2016), SA, jasmonic acid (JA), and a chemical compound paclobutrazol (PB) have been utilized as potential elicitors to enhance abiotic stress tolerance in plants (Pill and Gunter, 2001; Afzal et al., 2006; Bayat and Sepehri, 2012; Khan et al., 2013). In plant biology, elicitors are considered to be extrinsic or foreign molecules that act at very low concentrations and induce plant defense responses. They can attach to specific receptor proteins located on plant cell membranes and are highly specific in elicit secondary metabolite production. Methyl jasmonate (MJ), a methyl ester of JA is a naturally produced plant hormone, which has been used to elicit plant’s defense response. Exogenous application of MJ increased antioxidant activity of plants under water stress (Bandurska et al., 2003) and treatment with MJ enhanced drought tolerance by increasing synthesis of biochemical compounds such as proline, ascorbic acid, soluble sugars and malondialdehyde (Nazarli et al., 2014). Recently, the role of JA as a critical regulator of proteasome degradation in response to abiotic stress in Arabidopsis and rice has been characterized (Wu et al., 2015). PB is a member of triazole family which have been characterized as plant protectants because of their capability to improve antioxidant potential in the stressed plants (Manivannan et al., 2007). It inhibits P450 monooxygenase enzyme and ent-kaurene oxidase which is involved in gibberellic acid biosynthesis. SA, being a plant hormone of phenolic nature and irrespective of its important role in biotic stress, mitigates the damaging effects of drought when applied at lower concentration. SA application suppressed production of reactive oxygen species (ROS) and enhanced the activity of superoxide dismutase and catalase in Kentucky Blue grass (Farooq et al., 2008). The synergistic and antagonistic actions of plant hormones with the drought-responsive abscisic acid (ABA) have been referred as signaling crosstalk (Fujita et al., 2006).

Sense of the stress induces signaling network in plant that activates ion channels, kinase cascades, production of ROS, accumulation of hormones such as ABA, ethylene, JA and SA (Pérez-Clemente et al., 2012). Plants produce MJ during drought stress, which in turn stimulates the production of ABA. Drought-inducible genes encoding the key enzymes of ABA biosynthesis pathway, cellular protective enzymes, signaling proteins and transcription factors (TFs) have been identified in Arabidopsis, rice, and other plant species (Zhu, 2002). Rice *OsJMT1* and *OsSDR* genes were found to be involved in MJ and ABA biosynthesis, respectively. Thus, plant responses to the stress are mediated via changes in gene expression which result in alteration in transcriptome, proteome and metabolome. Studies have been conducted in different plant species to investigate the underlying mechanism of priming and stress memory (Ramirez et al., 2015; Wang et al., 2015). Recently, physio-biochemical and molecular mechanisms of seed-priming under drought, chilling and submergence stress in rice has been reported (Salah et al., 2015; Hussain et al., 2016a,b) which elaborate the importance of this technique. Still, much more strategic research is needed to combat drought stress in crop plants. ROS-induced protein oxidation may cause site-specific amino acid modifications, fragmentation of polypeptide, aggregation of cross-linked reaction products, altered electric charge, and increased susceptibility of proteins to proteolysis. In this context, plant-tissue contents of malondialdehyde (MDA) and carbonylated proteins are considered as the biochemical markers of lipid peroxidation (LP)/cell membrane damage and protein oxidation (Møller and Kristensen, 2004), respectively. Increased peroxidation (degradation) of lipids and the elevated protein oxidation have been found to be common in plants growing under environmental stresses (Mishra et al., 2011; Sharma et al., 2012). Measurement of protein oxidation on the basis of sulfhydryl group content indicated that increase in antioxidant enzymes (by SA and MJ) helps reducing protein oxidation, and in turn protects the plant from oxidative stress (Kumari et al., 2015).

Abscisic acid is well-known to regulate stress responses in plants through the expression of stress-responsive genes. Belda-Palazon et al. (2014) demonstrated that ABA controls production of hypusine (an unusual amino acid found in a eukaryotic translation elongation factor, eIF5A) in *Arabidopsis thaliana*. The hierarchically upstream regulators of the stress-responsive genes, mainly the TFs, directly control the expression of multiple genes by binding to the *cis*-regulatory elements in their promoter region. Thus, TFs are considered as master regulators of stress responses (Dey et al., 2016). APETALA2/ethylene-responsive element binding factor (AP2/ERF) family is a large group of plant-specific TFs that includes four major subfamilies: (i) AP2, (ii) Related-to-ABI3/VP1 (RAV), (iii) ERF, and (iv) DREB (dehydration-responsive element-binding protein). Many of the stress-inducible AP2, ERF and DREB subfamily members have been isolated and characterized. They can modulate transcription of downstream genes involved in drought tolerance (Mawlong et al., 2014). Regulation of transcription is a common target to modulate gene expression both under abiotic and biotic stresses.

The present study aimed at understanding physiobiochemical and molecular responses of contrasting rice genotypes at vegetative stage of rice plants raised from the seeds primed with either of the elicitors namely MJ, PB, or SA and grown under water-deficit stress. One of the objectives of the study was to investigate the possible use of the elicitor in mitigating the harmful effects of water-deficit stress, particularly in the drought-sensitive rice genotype.

## Materials and Methods

### Plant Material

Mature seeds of two contrasting rice genotypes Nagina-22 (N-22, drought-tolerant) and Pusa Sugandha-5 (PS-5, drought-sensitive) were obtained from the Division of Genetics, ICAR-Indian Agricultural Research Institute, New Delhi. Before priming, seeds of the contrasting rice genotypes were surface sterilized using 0.1% HgCl_2_ for 4 min and washed three times with sterilized double-distilled water.

### Experimental Details

In order to examine the role of seed-priming in alleviating the adverse effects of drought stress in rice, different concentrations of elicitor (MJ, PB, and SA) were tested in our preliminary study. The seeds were primed with each of the elicitor separately following the procedure described by Pill and Gunter (2001) for PB, Afzal et al. (2006) for MJ, and Khan et al. (2012) for SA. Based on the germination, seedling growth and vigor (fresh biomass), seed-priming with 100 μM was found to be most effective for enhancing drought tolerance in rice (Supplementary Figure S1 and Table S1), and this concentration was used in further experiments. The treatments used in the present study were (1) no-priming + no drought stress, (2) no-priming + drought stress, (3) MJ-priming + no stress, (4) MJ-priming + stress treated, (5) PB-priming + no stress, (6) PB-priming + stress treated, (7) SA-priming + no stress and (8) SA-priming + stress treated. Three seeds (primed or unprimed) were sown at equal distance in 4 × 4 inches pots, and grown in Phytotron facility under controlled conditions (12 h light, 30°C day/25°C night, 75% RH). Seedlings were irrigated with the half-strength Hoagland solution for 42 days, and then drought stress was imposed by withholding irrigation for 4 days while unstressed (control) seedlings were irrigated continuously. The experiment was laid out in completely randomized design with three biological and three technical replications. Shoot tissues from the biological and technical replicates were collected for biochemical and molecular studies.

### Estimation of Soil Moisture and Relative Water Contents

Soil moisture content (SMC) was determined by gravimetric method. Soil samples were collected from the 5 cm depth of the pots and placed in pre-weighed Petri plates. The weight of the soil was recorded immediately followed by drying at 60°C in an oven till constant/dry weight (DW) of the soil was obtained. SMC was calculated using the formula: SMC = [(weight of wet soil) - (weight of dried soil)]/(weight of dried soil).

Relative water content (RWC) in shoot was measured according to Barr and Weatherley (1962). Fresh leaves were cut at approximately one-third distance from the tip; a 10 cm piece was excised from the shoot and placed in a pre-weighed Petri-plate covered with a lid followed by taking fresh weight (FW) as soon as possible. The plate was then filled with distilled water, covered with lid and kept at room temperature for 4 h to achieve fully turgid condition. Turgid weight (TW) was taken, then leaves were blot dried and kept in an oven at 60°C for 24 h or until constant weight was obtained. DW was recorded and RWC was calculated using the formula: RWC (%) = [(FW-DW) / (TW - DW)] × 100

### Estimation of Total Phenolics Content

Total phenolics content (TPC) in the shoot samples of the contrasting rice genotypes was determined following the procedure described by Singleton et al. (1999). Briefly, 1.0 g of fresh shoot tissues were ground into fine powder to which 20 mL Trichloro acetic acid (0.1%) was added and the content was centrifuged at 12,000 rpm for 10 min at room temperature. The supernatant was collected and 0.5 mL of the aqueous extract was added to 2.5 mL of 10% Folin-Ciocalteu reagent (v/v) and 2 mL of 7.5% sodium carbonate. The reaction mixture was incubated at 45°C for 40 min and absorbance was recorded at 765 nm. Gallic acid was used as a phenol standard and TPC was expressed as mg of gallic acid equivalents g^-1^ extract.

### Level of Lipid Peroxidation

Lipid peroxidation was measured in terms of malondialdehyde (MDA) level in shoot tissues following the procedure described by Cakmak and Horst (1991), which is based on thiobarbituric acid reaction. The sample extract was obtained by grinding 1.0 g fresh tissues in 20 mL TCA (0.1%) solution followed by centrifugation at 12,000 rpm for 10 min. One mL supernatant was reacted with 4 mL TCA (20%) solution containing 0.6% thiobarbituric acid. The reaction mixture was heated for 30 min at 95°C, then cooled on ice followed by centrifugation at 12,000 rpm for 10 min. Absorbance of the supernatant was recorded at 532 and 600 nm, and MDA level was calculated using the extinction coefficient of 155 mM^-1^ cm^-1^ using the formula: MDA level (nM) = ΔA_(532 nm - 600 nm)_ /1.56 × 10^5^.

### Total Antioxidant Activity

Total antioxidant activity (AO) in the shoot tissue extracts was measured using the stable DPPH radical following the method described by Hatano et al. (1988). Fresh tissues (1.0 g) were ground into fine powder and extracted with 10 mL ethanol (90%) by constant shaking at room temperature for 48 h. The extract was centrifuged 13,000 rpm for 10 min and the supernatant was used for estimation of antioxidant activities. The alcoholic solution of DPPH radical (0.5 mL, 0.2 mM) was added to 100 μL of the sample extract, mixed vigorously and kept standing in dark for 40 min. Then absorbance was measured at 517 nm and the capacity to scavenge DPPH radical was calculated using the formula: Scavenging (%) = [(A_0_ - A_1_) /A_0_)] × 100; where A_0_ is absorbance of the control reaction and A_1_ is absorbance of the sample at 517 nm. Inhibitory concentration at 50% (extract concentration that cause 50% scavenging of DPPH radical, IC_50_) was also determined.

### Level of Protein Oxidation

To measure the level of protein oxidation (PO), the sulfhydryl (SH) group of proteins was determined using Ellman’s reagent [5,5′-dithiobis(2-nitrobenzoic acid)] following the procedure described by Ellman (1959). Shoot tissues (75 mg) were ground into fine powder and suspended in 10 mL phosphate buffer (0.1 M, pH 8.0) containing EDTA (1 mM) and SDS (1%). The suspension was kept for stirring at 20°C for 30 min. For SH determinations, 3 mL protein extract was added with 3 mL of 0.l M phosphate buffer containing EDTA, SDS, and 0.1 mL Ellman’s reagent. The content was, mixed thoroughly by vortexing and incubated at 25°C for 1 h. The mixture thus obtained was centrifuged at 10,000 rpm for 30 min and absorbance of the supernatant was measured at 412 nm against the reagent blank. Calculation for SH group was based on extinction coefficient of 13,600 M^-1^cm^-1^ for thiolate-chromogen using the equation: μM SH g^-1^ = 73.53 × A_412 nm_ × D/C, where A_412_ = absorbance at 412 nm, C = sample concentration in mg solids mL^-1^, D = dilution factor (2.03) for SH, and 73.53 is derived from 10^6^/(13,600); 10^6^ is for conversion from the molar basis to μM mL^-1^ basis and from mg solid to g solid.

### Semi-quantitative Gene Expression Analysis

To assess the effects of seed-priming and drought stress on expression of *R*ice *D*rought-responsive genes (*RD1*: EF362638 and *RD2*: KC988330), total RNA was isolated from shoot tissues of the contrasting rice genotypes under different treatments. RNA isolation, complimentary DNA (cDNA) synthesis and reverse transcription PCR (RT-PCR) were carried out with the gene-specific primers [*RD1*: FP 5′-TCGGCTGCGAAGGTGGCAGC-3′, RP 5′- CACCGTGCAGCAGCCCATGT-3′); *RD2*: FP 5′-CGCCTCAAAGAAGCGCTAC-3′, RP 5′-AGTGTCAAAGGTGCCAAGC-3′] following the procedure described elsewhere (Singh et al., 2015). Actin (UniGene cluster Os03G50885) was used as housekeeping/reference gene (FP 5′-GATCTGGCATCACACCTTCTAC-3′, RP 5′-CTGGGTCATCTTCTCACGATTG-3′). Amplification products were analyzed on agarose (1.4%) gel electrophoresis.

### Quantitative Gene Expression Analysis

To study differential expression of the *RD1* and *RD2* genes under different treatments, quantitative expression analysis of the genes was carried out following the MIQE guidelines and the protocol mentioned earlier (Singh et al., 2015). Three biological and three technical replications were used for the analysis. RT-qPCR was performed in 20 μL reaction mix on Mx3000PTM Real Time PCR system (CFX-96, Bio-Rad Platform) with SYBR Green qPCR Master Mix kit (Bio-Rad; Cat. # 170-8880AP) using gene-specific primers. The thermal cycler was programmed for an initial denaturation at 95°C for 3 min, followed by 39 cycles each of 20 s denaturation at 94°C, 20 s annealing at 60°C and 40 s extension at 72°C. Amplification data collection was set at the end of each extension step. The data was analyzed through melt curve analysis to check the specificity of PCR amplification. Pfaffl formula (Ratio = 2^-ΔΔCt^) [where ΔΔCt = (ΔCt sample-ΔCt control); ΔCt sample = (ΔCt target-ΔCt reference) for all primed and unprimed samples; and ΔCt control = (ΔCt target-ΔCt reference)] (Pfaffl, 2001) was used to calculate the relative expression of the *RD1* and *RD2* genes with actin (UniGene cluster Os03G50885) as reference/housekeeping gene.

### Statistical Analysis

All the experiments were carried out in triplicate. The data were analyzed with the help of pre-loaded software in Excel, programmed for statistical calculations. Duncan’s multiple range tests (DMRT) were performed to determine significant difference between means at a significance level of *P* ≤ 0.05 and reported as the mean ± standard deviation (SD).

## Results

### Seed-Priming Mitigates Damaging Effects of Drought Stress on the Plant

**FIGURE 1:**
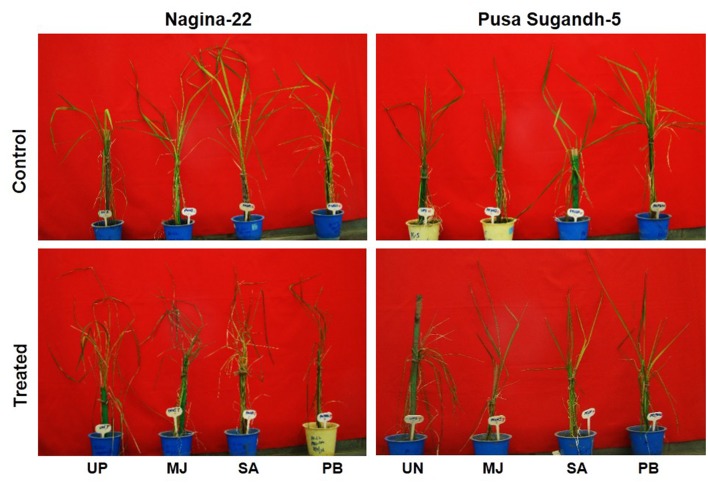
Effect of seed-priming on mitigation of the deleterious effects of drought stress (withholding irrigation for 4 days) in 7 week old seedlings of Nagina-22 (N-22, drought-tolerant) and Pusa Sugandh-5 (PS-5, drought-sensitive) rice genotypes primed with 100 μM of methyl jasmonate (MJ), salicylic acid (SA), or paclobutrazol (PB). UP, unprimed + no drought stress; MJ, MJ-priming; PB, PB-priming; SA, SA-priming.

Seed-priming with elicitor (MJ, SA or PB) showed beneficial effects on morphological features of the seedlings of drought-tolerant (N-22) and drought-sensitive (PS-5) rice genotypes under drought stress. The plants raised from primed-seeds showed healthier growth under the stress compared to those raised from unprimed seeds. Physiological health of the plants raised from unprimed seeds of drought-tolerant (N-22) and drought-sensitive (PS-5) genotypes under control as well as drought stress condition was found to be poorer compared to that of the plants raised from the seeds primed with any of the elicitor (**Figure 1**). Comparative morphological observations on the plants revealed that response of the elicitors was better in case of the drought-sensitive genotype than that observed in the drought-tolerant genotype. MJ and PB were found to be more responsive than SA on seed-priming, providing better protection against the drought-stress in both the rice genotypes.

### Seed-Priming Increased Relative Water Content of Shoot under Drought Stress

**FIGURE 2:**
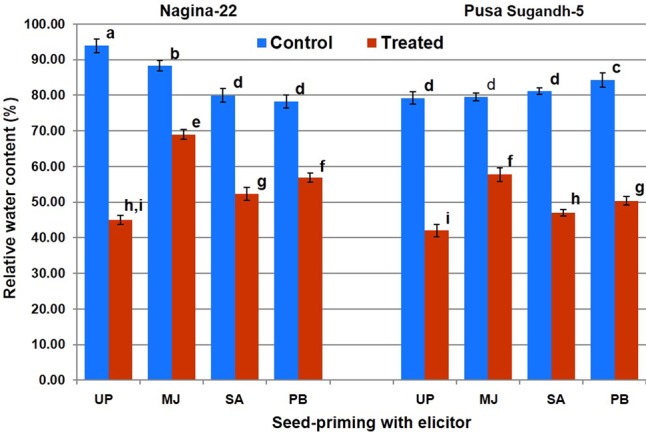
Effect of seed-priming on relative water content in shoot of the contrasting rice genotypes [Nanina-22 (N-22, drought-tolerant) and Pusa Sugandh-5 (PS-5, drought-sensitive)] under control and drought stress conditions. UP, unprimed + no drought stress; MJ, MJ-priming; PB, PB-priming; SA, SA-priming. Columns with different lowercase letters indicate significant difference at *P* < 0.05 (Duncan’s multiple range test). Bar represents the standard deviation.

Measurement of the SMC in the pots on 0, 2, 4, and 6 day of withholding irrigation indicated that a progressive decline in SMC occurred with the progress in the duration of drought stress (Supplementary Table S2). Initially, SMC of the pots was in the range of 50–65% while it was reduced to 26–36% on 4th day, and 18–24% on 6th day of the water-deficit stress. We observed that the stress of more than 4 days led to the threshold level; therefore, shoot tissues were collected after 4 days of the stress for the biochemical and molecular studies. RWC in shoot was found in the range of 80–96% in the plants of both the genotypes raised from primed and unprimed seeds under control (unstressed) condition (**Figure 2**). Drought stress caused decrease in the RWC of shoot of the plants from both primed and unprimed seeds. The decrease in RWC was significantly higher (56–58%) in case of the plants from unprimed seeds than that (22–52%) in the plants from primed seeds of the contrasting rice genotypes. RWC of the plants from MJ-primed seeds under the stress was found to be maximum (70 and 58%), followed by the plants from PB-primed (58 and 52%) and the plants from SA-primed (56 and 45%) seeds of drought-tolerant and drought-sensitive genotypes, respectively. Thus, among the three elicitors used for seed-priming, reduction in RWC due to drought stress was found to be lowest in the plants from MJ-primed seeds (N-22, 21% and PS-5, 27%).

### Seed-Priming Increased Antioxidant Activities in Shoot under the Stress

**FIGURE 3:**
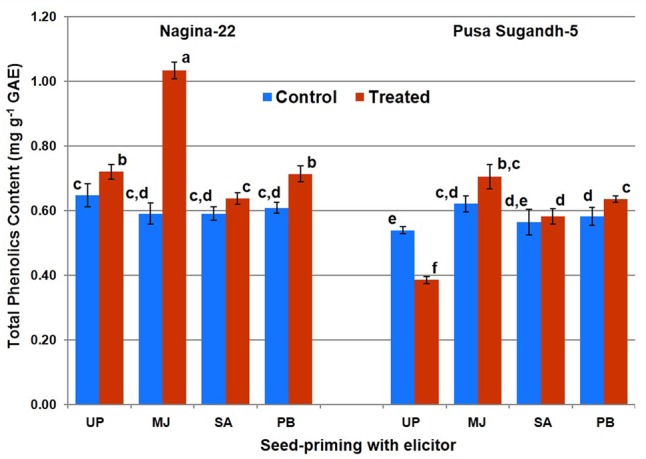
Effect of seed-priming on total phenolic content in shoot of the contrasting rice genotypes [Nanina-22 (N-22, drought-tolerant) and Pusa Sugandh-5 (PS-5, drought-sensitive)] under control and drought stress conditions. Columns with different lowercase letters indicate significant difference at *P* < 0.05 (Duncan’s multiple range test). Bar represents the standard deviation.

Total phenolics content was found to increase (10%) on drought stress imposition in the drought-tolerant (N-22) genotype, while it was found to decrease (27%) in the drought-sensitive (PS-5) genotype (**Figure 3**). Increase in TPC due to drought stress was found to be 45% in the plants raised from MJ-primed seeds of N-22, while the increase was 15% in the plants raised from MJ-primed seeds of PS-5. Significant increase in TPC on drought stress imposition in both the genotypes was also observed on seed-priming with PB, while the increase was non-significant in the plants raised from the seeds primed with SA.

**FIGURE 4:**
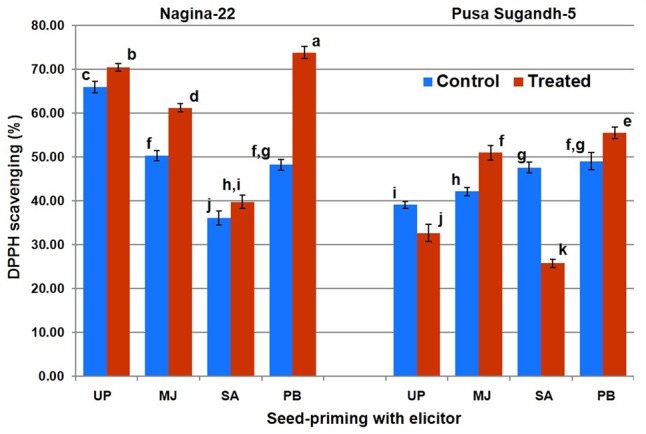
Effect of seed-priming on antioxidant activity (DPPH scavenging %) in shoot of the contrasting rice genotypes [Nanina-22 (N-22, drought-tolerant) and Pusa Sugandh-5 (PS-5, drought-sensitive)] under control and drought stress conditions. UP, unprimed + no drought stress; MJ, MJ-priming; PB, PB-priming; SA, SA-priming. Columns with different lowercase letters indicate significant difference at *P* < 0.05 (Duncan’s multiple range test). Bar represents the standard deviation.

Antioxidant potential in terms of DDPH scavenging was found to increase (7.5%) on drought stress imposition in the drought-tolerant (N-22) genotype, while it was found to decrease (20%) in the drought-sensitive (PS-5) genotype (**Figure 4**). Increase in antioxidant potential due to drought stress was found to be 22% in the N-22 plants raised from MJ-primed seeds, while the increase was 21% increase in the PS-5plants raised from MJ-primed seeds. Seed-priming with PB also showed significant increase in antioxidant potential on drought stress imposition in N-22 (51%) and PS-5 (12%), but seed-priming with SA caused decrease (54%) in antioxidant potential in drought-sensitive genotype under the stress.

### Seed-Priming Lowered Lipid Peroxidation in Shoot under the Stress

**FIGURE 5:**
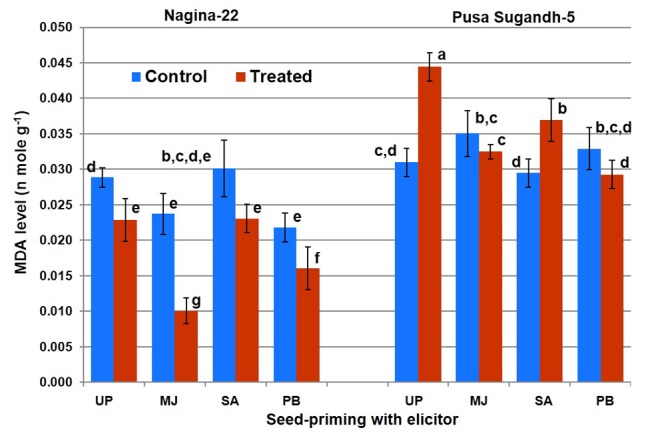
Effect of seed-priming on lipid peroxidation (MDA level) in shoot of the contrasting rice genotypes [Nanina-22 (N-22, drought-tolerant) and Pusa Sugandh-5 (PS-5, drought-sensitive)] under control and drought stress conditions. UP, unprimed + no drought stress; MJ, MJ-priming; PB, PB-priming; SA, SA-priming. Columns with different lowercase letters indicate significant difference at *P* < 0.05 (Duncan’s multiple range test). Bar represents the standard deviation.

Lipid peroxidation in terms of MDA level was found to decrease (21%) in the drought-tolerant (N-22) genotype on drought stress imposition, while it was found to increase (45%) in the drought-sensitive (PS-5) genotype (**Figure 5**). Seed-priming with the elicitors lowered LP on drought stress imposition in the shoot of both the genotypes. However, SA-priming was less efficient in mitigating LP in drought-sensitive (PS-5) genotype under the stress. Decrease in LP in N-22 under drought stress due to seed-priming with MJ was up to 58%, while the decrease was 27% due to seed-priming with PB. In case of drought-sensitive (PS-5) genotype, the decreased LP under drought stress due to seed-priming with MJ and PB was non-significant, while seed-priming with SA caused increase (23%) in LP under drought stress.

### Seed-Priming Reduced Protein Oxidation in Shoot under the Stress

**FIGURE 6:**
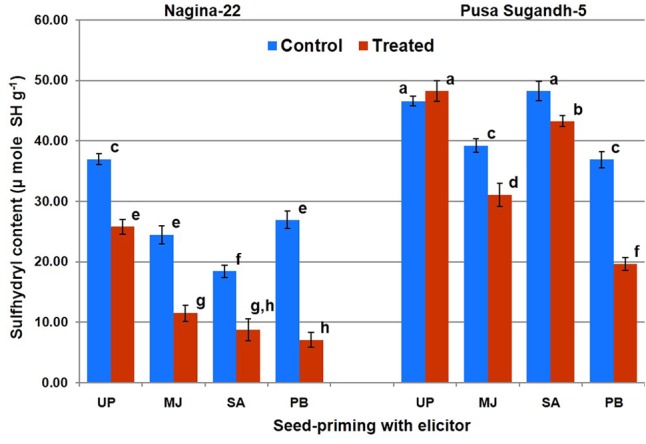
Effect of seed-priming on protein oxidation (sulfhydril content) in shoot of the contrasting rice genotypes [Nanina-22 (N-22, drought-tolerant) and Pusa Sugandh-5 (PS-5, drought-sensitive)] under control and drought stress conditions. UP, unprimed + no drought stress; MJ, MJ-priming; PB, PB-priming; SA, SA-priming. Columns with different lowercase letters indicate significant difference at *P* < 0.05 (Duncan’s multiple range test). Bar represents the standard deviation.

Protein oxidation (PO) was found to be significantly higher in drought-sensitive (PS-5) genotypes compared to that in the drought-tolerant (N-22) genotype. Seed-priming with the elicitor reduced level of PO under the stress in both the genotypes (**Figure 6**). Priming with PB showed maximum decrease in the level of PO, followed by MJ and SA in both the genotypes after drought stress imposition. PB primed drought treated sample showed maximum (73%) decrease in PO level in the tolerant genotype, and up to 46% decrease in the sensitive genotype compared to the PO level in plants raised from unprimed seeds and grown under the stress. MJ treatment caused reduction in PO by 51 and 20% in the tolerant genotype and 46% decrease in sensitive genotype, respectively. SA showed 50% decrease in PO in case of tolerant-genotype under the stress, while it was less effective in reducing PO in the drought-sensitive genotype.

### Seed-Priming Upregulated Expression of the *RD* Genes under the Stress

**FIGURE 7:**
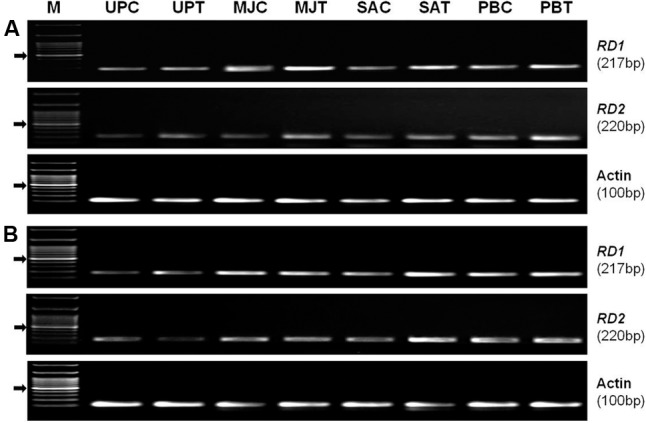
Semi-quantitative (RT-PCR) expression profile of *RD1* and *RD2* genes in shoot of the plants raised from the seeds of contrasting rice genotype **(A)** Nanina-22 (N-22, drought-tolerant) and **(B)** Pusa Sugandh-5 (PS-5, drought-sensitive) without priming or priming with different elicitor grown under control and drought stress (witholding irrigation for 4 days) conditions. Actin was used as reference gene. UPC: unprimed, unstressed (control); UPT: unprimed, stressed (treated with stress); MJC: MJ-primed, control; MJT: MJ-primed, treated; SAC: SA-primed, control; SAT: SA-primed, treated; PBC: PB-primed, control; PBT: PB-primed, treated.

Semi-quantitative (RT-PCR) expression analysis of *RD1* (EF-362638) and *RD2* (KC-988330) genes from AP2/ERF TF family, in shoot of the plants raised from the seeds of the contrasting rice genotype (N-22, drought-tolerant and PS-5, drought-sensitive) primed with an elicitor (MJ, SA, or PB) showed that the genes were differentially expressed under control and drought stress conditions (**Figure 7**). Seed-priming was found to upregulate expression level of the genes in shoot of both the genotype under drought stress. In the drought-tolerant genotype under the stress, the increase in expression level of *RD1*gene due to seed-priming with MJ was more prominent than that with PB and SA. Whereas, the increase in expression level of *RD2* gene in the drought-tolerant genotype under the stress was more prominent due to seed-priming with PB (**Figure 7A**). In case of drought-sensitive genotype under the stress, the increase in expression level of both the genes were found to be more due to seed-priming with SA and PB than that due to seed-priming with MJ (**Figure 7B**). Expression level of the housekeeping/reference (Actin) gene was observed to be constant in primed and unprimed, control and stressed samples of both the rice genotypes.

**FIGURE 8:**
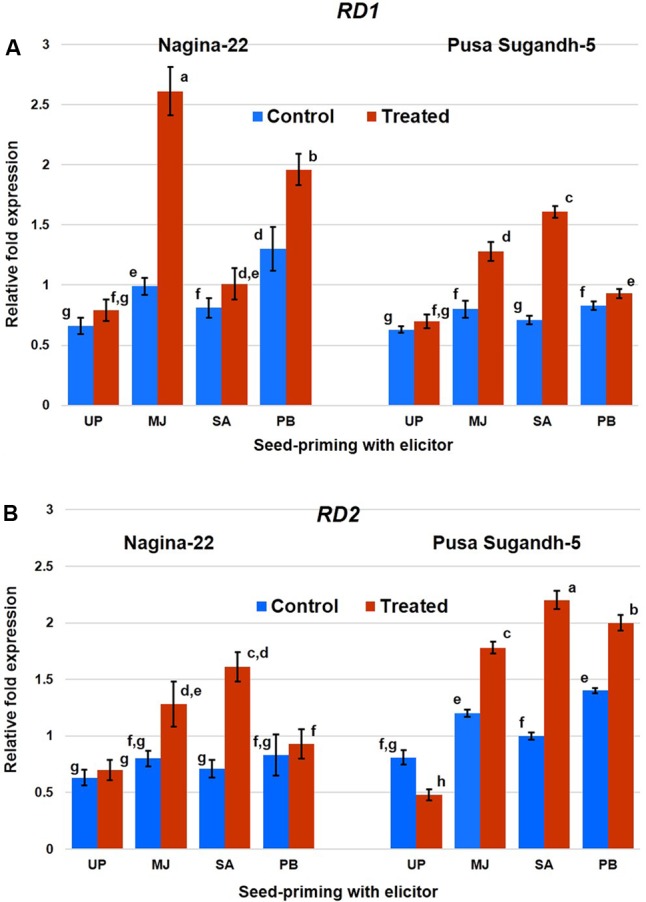
Quantitative (RT-qPCR) expression profile of **(A)** *RD1* and **(B)** *RD2* genes in shoot of the plants raised from the seeds of contrasting rice genotypes (Nanina-22, drought-tolerant and Pusa Sugandh-5, drought-sensitive) without priming or priming with different elicitor grown under control and drought stress (witholding irrigation for 4 days). The results are represented as mean fold change in relative expression over three biological and three technical replicates, normalized with respect to actin as housekeeping/reference gene. UP, unprimed; MJ, MJ-primed; SA, SA-primed; PB, PB-primed. Columns with different lowercase letters indicate significant difference at *P* < 0.05 (Duncan’s multiple range test). Bar represents the standard deviation.

Quantitative (RT-qPCR) expression analysis of *RD1* and *RD2* genes in shoot of the rice plants raised from the seeds of the contrasting rice genotype primed with the elicitor clearly showed differential expression of the genes under control and drought stress conditions (**Figure 8**). Expression level of the genes was found to be upregulated due to the stress in drought-tolerant genotype, while it was found to be downregulated in the drought-sensitive genotype. Seed-priming with MJ significantly increased expression of the genes even under control condition, and drought stress further increased the expression of these genes. MJ induced expression level of the *RD1* gene in N-22 under drought stress by 2.6-fold, while 1.6-fold in case of PS-5. Seed-priming with PB also increased expression level of the gene significantly (2-fold) in N-22 under drought stress, but the increase in expression level of the gene due to SA-priming was found to be less compared to that caused by MJ and PB priming. Expression of *RD2* was found to be increased significantly under drought stress due to seed-priming with the elicitors. It was found to be maximum (3.7-fold) on priming with PB in case of N-22, and 2.2-fold in case of PS-5 on SA-priming. Comparative analysis indicated that the effect of seed-priming was more influential on expression of the gene in the drought-sensitive genotype (converting its downregulated expression to upregulated expression under the stress) than that in the drought-tolerant genotype.

## Discussion

Most of the rice varieties are severely affected by abiotic stresses (Almeida et al., 2016). Among the abiotic stresses, drought stress alone or in combination with other abiotic stress(es) causes severe damages to the crop plants, which result in reduced growth, development and productivity of the crop. Our morphological observations clearly indicated that the two contrasting rice genotypes (N-22, drought-tolerant and PS-5, drought-sensitive) displayed distinct variation in growth under drought stress during vegetative growth. The observed reduction in the growth might be due low osmotic potential as well as due to the decrease in cell wall extensibility (Grieve et al., 2001; Haplerin and Lynch, 2003). Despite of very low SMC (25–35%), MJ was found to be more effective in maintaining high RWC (58–69%) in shoot compared to that (42–45%) in the plants raised from unprimed seeds under drought stress in both drought-tolerant and drought-sensitive genotypes. The effects of seed-priming could be correlated with the better health and growth of the plants under drought stress compared to that of the plants from unprimd seeds (**Figure 1**). Seed-priming with different elicitor could significantly (12–53%) increase RWC in shoot under the stress compared to that in the plants from unprimd seeds. Recently, the effect of drought stress on shoot RWC was reported to be associated with the tuber yield in potato (Soltys-Kalina et al., 2016). RWC is considered as a useful indicator of plant health status under drought stress (Chaves et al., 2002; Mawlong et al., 2014), and shows relationship with yield parameters (Gutierrez et al., 2010).

Seed-priming with PB caused significant increase in TPC on drought stress imposition in both the genotypes, while MJ showed significant increase in case of the tolerant genotype (**Figure 2**). Seed-priming with MJ and PB was found to increase AO activity in drought-sensitive genotype which may improve drought tolerance ability of the genotype. On the contrary, we observed SA to decrease AO activity by 45% in the drought-sensitive genotype (**Figure 4**), which might be acting as antagonist (Jisha et al., 2015). A positive correlation between TPC and AO activity has been reported earlier to impart abiotic stress tolerance in plants (Trust et al., 2005; Rao et al., 2013). Thus, the contrasting genotypes responded differently (in terms of their antioxidant potential) to seed-priming with different elicitor. Though seed-priming with SA could not increase TPC content (positively correlated with drought stress tolerance) significantly in both the genotypes (**Figure 3**), it caused significant reduction in PO (negatively correlated with drought stress tolerance) under the stress (**Figure 6**). We observed reduced accumulation of MDA in drought-tolerant (N-22) genotype compared to that in the drought-sensitive (PS-5) in the plants raised from unprimed seeds under the stress. MDA level increases under drought stress due to peroxidation of unsaturated fatty acids in phospholipids, and LP has been used as an indicator of free radical damage to cell membrane under stress conditions (Jaleel et al., 2007). Seed-priming with SA did not show beneficial effect on MDA level (negatively correlated with drought stress tolerance) in case of drought-tolerant rice genotype, but significant increase in MDA level was observed in case of the drought-sensitive genotype (**Figure 5**). To overcome the deleterious effects of oxidative stress, plants make use of a complex antioxidant systems and accumulate phenolic compounds to minimize the damages caused by the free radicals resulting into lower LP and cell membrane damages. Farooq et al. (2008) and Khan et al. (2012) reported beneficial effects of seed-priming in terms of better germination, more vigorous growth, early flowering and higher yield. In the present study, we observed significant retardation in the growth and development of contrasting rice genotypes under drought-stress which is in agreement with the observations reported earlier (Afzal et al., 2006; Bhargava et al., 2015). It may be due to the reduced water potential, increased reactive oxidant potential and membrane damages. Salah et al. (2015) and Hussain et al. (2016a,b) also described the significance of seed-priming in mitigating drought, chilling and submergence stresses in rice.

Transcription factors play crucial role in response of the plants to abiotic stresses. Genome-wide analysis of AP2/ERF genes and their expression in rice has been reported to play important role in stress tolerance (Tang et al., 2016). We observed differential expression of *RD1* and *RD2* genes in shoot of the contrasting rice genotype (**Figure 8**). The genes were found to be upregulated under the stress in drought-tolerant genotype while it was downregulated in the drought-sensitive genotype. Seed-priming with elicitor significantly increased expression level of both the genes, particularly under drought stress in the drought-sensitive genotype. However, expression of *RD2* gene was more significantly induced [from downregulated expression in the drought-sensitive genotype (PS-5) under the stress] to more than 2-fold upregulated expression due to seed-priming under the stress. Recently, Wei et al. (2016) reported ectopic expression of a TF (DREB1A) in Arabidopsis showing higher RWC, chlorophyll content, photosynthetic rate, increased superoxide dismutase, catalase, peroxidase activities and lower MDA level when subjected to drought stress. All of these resulted into increased drought tolerance in the transgenic lines.

**FIGURE 9:**
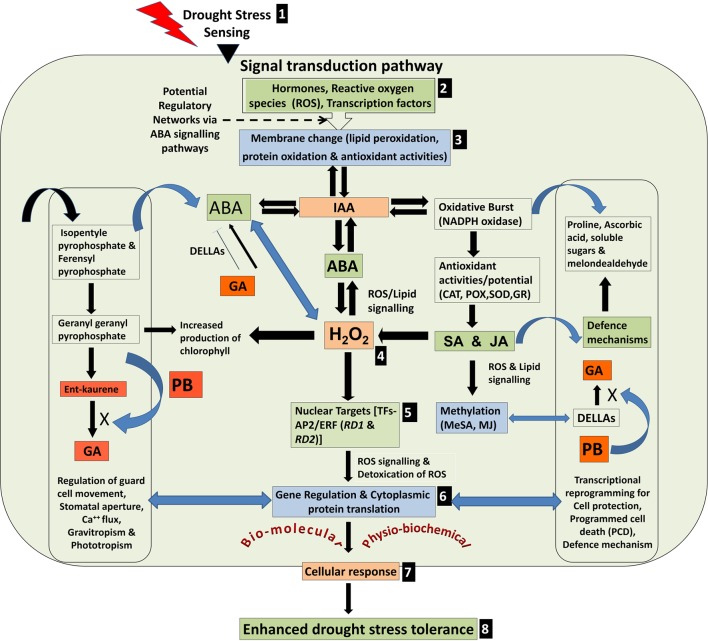
Model depicting various possible physio-biochemical and bio-molecular responses under drought stress. **(1)** Stress sensing, **(2)** Signal transduction through various inducers, **(3)** Membrane alterations, **(4)** Hydrogen peroxide accumulation (H_2_O_2_) through potential regulatory networks, **(5)** Induction of AP2/ERF transcription factor genes (*RD1* and *RD2*), **(6)** Transcriptional and translational reprogramming to combat the stress, **(7)** cellular responses, and **(8)** enhanced drought tolerance.

A model depicting various possible physio-biochemical and bio-molecular responses in plant cell under drought stress is presented in **Figure 9**. The stress is sensed through cell membrane, transduced to various inducers to regulate structural and molecular alterations including hydrogen peroxide accumulation, induction of AP2/ERF TF genes and transcriptional/translational reprogramming for protective defense mechanism. We observed a better response of MJ followed by PB to induce the defense responses of rice against drought stress. The effects of seed-priming were beneficial for growth and development of the rice plants not only under the stress but also under control condition in the plants raised from primed seeds (**Figure 1**). This indicated that seed-priming with elicitor did not adversely affected metabolic pathways in the plants under normal (unstressed) condition. In turn, it lays foundation toward the development of an easier, efficient and economic technique toward improving drought stress tolerance in rice. Characterization of gene encoding jasmonic acid carboxy methyl transferase has provided information on the role(s) of phytohormone in gene activation and systemic long-distance signaling (Cheong and Choi, 2003). Salzman et al. (2005) and Jisha et al. (2015) reported transcriptional cross-talk between SA and MJ indicating their antagonism and synergism in gene regulation and signaling cascade.

Transgenic rice plants overexpressing AP2/ERF TF (*OsEREBP1*) showed increase in endogenous levels of α-linolenate, several jasmonate derivatives and ABA but not SA. The level of ABA was ∼2-fold higher in the transgenic plants as compared to non-transgenic control, whereas SA levels showed no significant difference (Jisha et al., 2015). By contrast, we observed that seed-priming with SA caused maximum increase in the expression of *RD1* and *RD2* in drought-sensitive genotype under drought stress. But in case of drought-tolerant genotype, *RD1* did not show significant increase while *RD2* showed ∼2-fold increase under the stress. It suggests that function of SA is ambiguous because at physio-biochemical level it acts as an antagonist, while synergistic at gene expression level. Lipids are found to be connected to the plant defense responses through potential action as a signaling molecules (Laxalt and Munnik, 2002) through the fatty acid- linolenic acid, acting as the precursor for 12- oxo-phytodienoic acid (OPDA) and JA synthesis via octadecanoid pathway (Creelman and Mullet, 1997). Suppression of plant growth by PB occurs because it blocks three separate steps (Geranylgeranyl pyrophosphate → Ent-kaurene → GA) in the terpenoid pathway for the production of gibberellins which diverts isopentyle pyrophosphate and ferensyl pyrophosphate (secondary metabolites) toward ABA biosynthesis (**Figure 9**).

Findings here indicated that RWC, total phenolic content (TPC), LP, protein oxidation and antioxidant (AO) activity are crucial biochemical markers of drought tolerance. Both *RD1* and *RD2* genes were upregulated under drought stress, and seed-priming with MJ, PB and SA further increased expression of both of the genes of AP2/ERF TFs. There was a progressive increase in TPC, LP, AO and protein oxidation activity in primed samples during drought stress imposition and increase was found to be of higher magnitude in the drought-susceptible genotype than that of the tolerant one. This indicates that elicitor enhances drought tolerance and plays important role in eliciting defense mechanisms against abiotic stress. Seed-priming enhanced drought tolerance in drought-sensitive genotype at much higher level than that in drought-tolerant rice genotype. The elicitors were found to mitigate the effect of drought stress by affecting expression of *RD1* and *RD2* genes as well affecting biochemical and physiological parameters. The great leaps and bounds toward understanding plant’s drought stress responses and tolerance mechanisms have been achieved in the past years; however, many challenges still lie ahead and plant breeders face serious difficulties in developing superior cultivars with broad spectrum of abiotic resistance. The regulation of gene expressions and signaling cascades that regulate defense mechanism remain to be elucidated. Thus, present findings will facilitate insight in unraveling elicitor induced biochemical and molecular manifestations of drought tolerance in rice to improve crop yield.

## Conclusion

Our findings confirmed that RWC, TPC, LP, PO and AO activity are important biochemical markers of drought stress tolerance in plants. A progressive increase in TPC and AO activities, while decrease in LP and PO were observed under drought stress in the plants raised from the seeds primed with elicitor (MJ or PB). The increase was found to be of higher magnitude in the drought-sensitive genotype (PS-5) than that of the drought-tolerant one. The drought-responsive (*RD1* and *RD2*) genes of AP2/ERF TF family were upregulated on drought stress, and seed-priming with the elicitors increased expression of the genes in the contrasting rice genotypes. This indicates that elicitor may play important role in mitigating abiotic stress in plants, particularly in the sensitive genotypes. Considerable progress has been made toward understanding the stress responses and tolerance mechanisms in plants in the past (Zhu, 2016). This indicates that seed-priming with elicitor does not adversely affect metabolic pathways in the plants under normal (unstressed) condition. In turn, it lays foundation toward the development of an easier, efficient and economic technique toward improving drought stress tolerance in rice. However, there is need to validate the efficacy of the seed-priming technique for improving yield of rice under drought stress in the field conditions. Our results also suggest that genetic manipulation of *RD1* and *RD2* genes of the AP2/ERF family may enhance production of secondary metabolites resulting in improved drought stress tolerance (Mawlong et al., 2015). Epigenetic regulation of gene expression (Wang et al., 2016) under abiotic stresses (Kumar and Singh, 2016; Kumar et al., 2017) and signaling cascades (Bernsdorff et al., 2016; Ji et al., 2016) that regulate defense mechanisms remain to be elucidated. The present findings would facilitate unraveling the elicitor induced biochemical and molecular manifestations of drought tolerance in crop plants to improve the yield.

## Author Contributions

AS and SK conceptualized, initiated and designed the research work, and wrote the manuscript; MKS, MS, and MA carried out the biochemical and molecular studies, data collection and their analysis; AT and SAM helped in critical assessment and execution of experimental plan, OY helped in maintaining experimental materials, conducting the experiments under controlled conditions, sample collection, etc.

## Conflict of Interest Statement

The authors declare that the research was conducted in the absence of any commercial or financial relationships that could be construed as a potential conflict of interest.
