# Supplementary material for: Elicitor-Induced Biochemical and Molecular Manifestations to Improve Drought Tolerance in Rice (Oryza sativa L.) through Seed-Priming
Source: Front Plant Sci. 2017 Jun 6;8:934. doi: 10.3389/fpls.2017.00934 (PMC5459913; doi:10.3389/fpls.2017.00934)
Supplement: Supplementary file 1 [file Data_Sheet_1.docx]

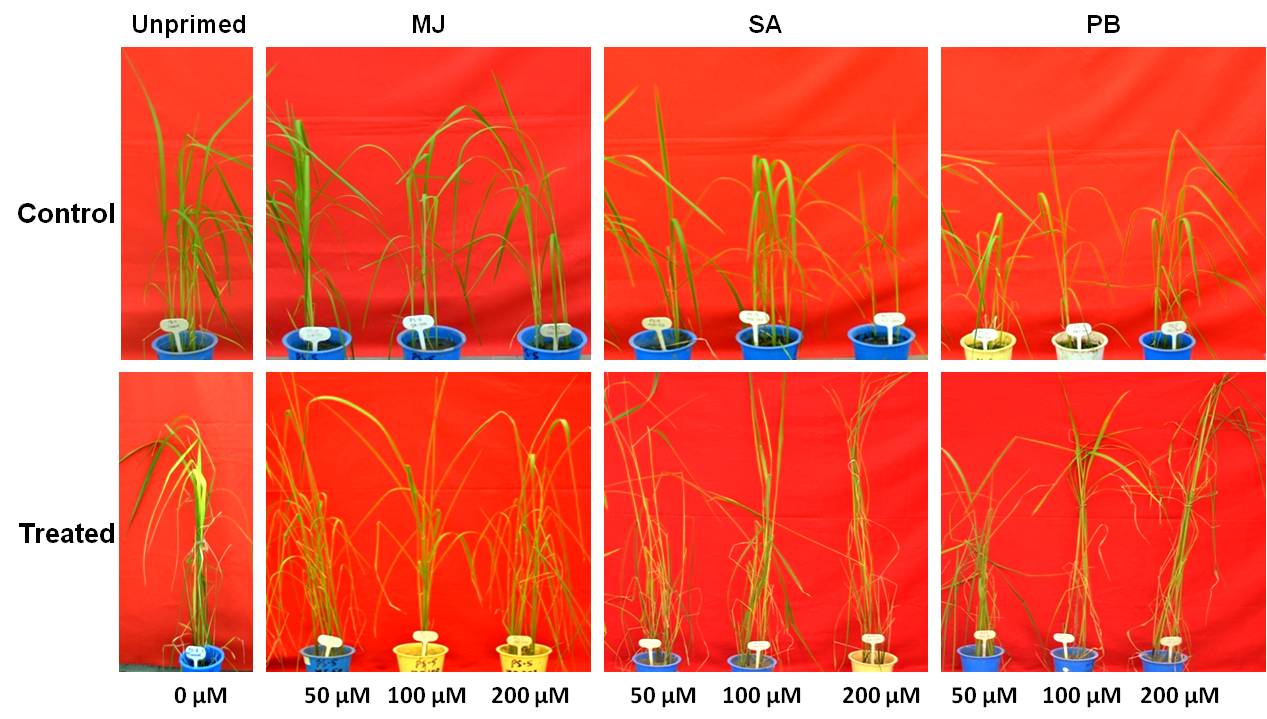

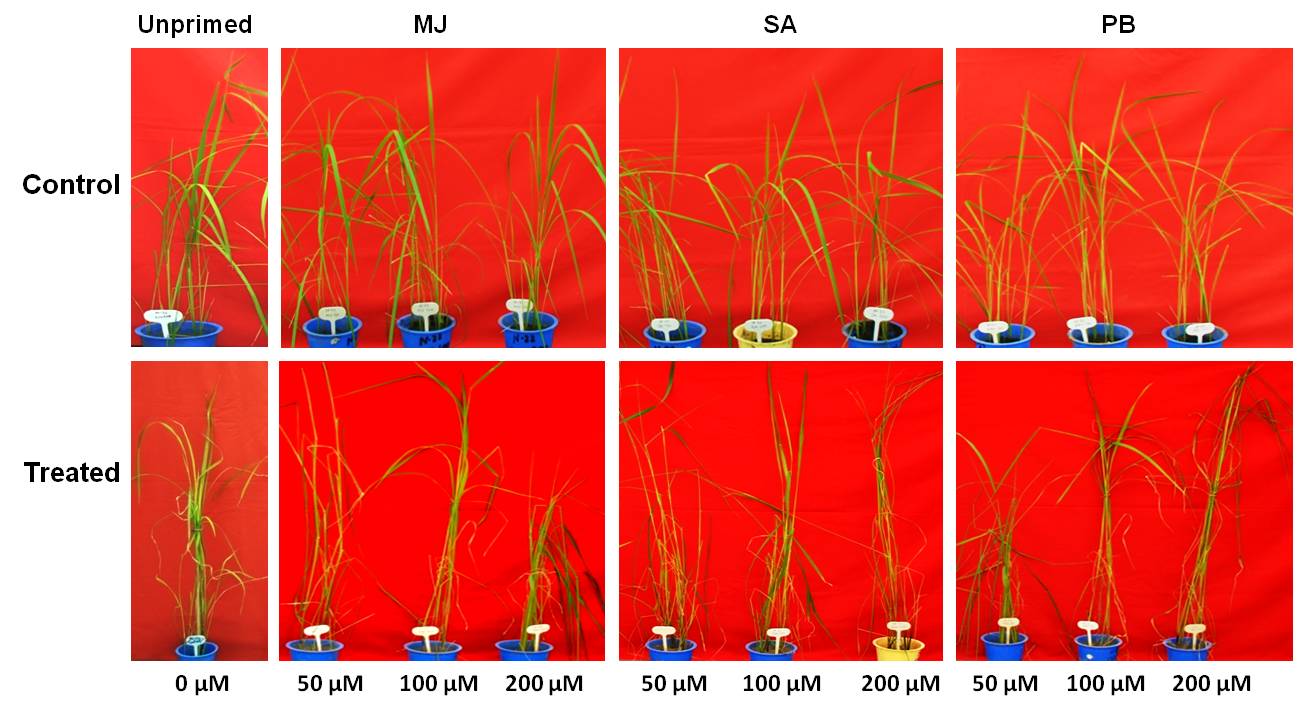


**b**

**a**

**Supplementary Figure S1**. Morphological features of (**a**) Nagina-22 (drought-tolerant) and (**b**) Pusa Sugnadh-5(drought sensitive) rice genotypes primed with methyl jasmonate (MJ), salicylic acid (SA) and paclobutrazol (PB) photographed after 4 days of drought stress imposition.

**Supplementary Table S1.** Effect of seed priming with different concentration (50 µM, 100 µM, 200 µM) of bioelicitors [methyl jasmonate (MJ), salicylic acid (SA), paclobutrazol (PB)] on vegetative growth of contrasting rice genotypes: Nagina-22 (drought-tolerant) and Pusa Sugandh-5 (drought-sensitive) under drought stress.

| **Sampl^*^** | **Bioelicitor concentration** | | | | | | | | | | | | | | |
| --- | --- | --- | --- | --- | --- | --- | --- | --- | --- | --- | --- | --- | --- | --- | --- |
|  | **0 µM** | | |  | **50 µM** | | |  | **100 µM** | | |  | **200 µM** | | |
|  | **Height**  **(cm)** | **Tillers**  **(#)** | **FB^Ψ^**  **(g)** |  | **Height**  **(cm)** | **Tillers**  **(#)** | **FB^Ψ^**  **(g)** |  | **Height**  **(cm)** | **Tillers**  **(#)** | **FB**  **(g)** |  | **Height**  **(cm)** | **Tillers**  **(#)** | **FB^Ψ^**  **(g)** |
| UPCN | 49^a^ | 7 | 136^a,1^ |  | **-** | **-** | **-** |  | **-** | **-** | **-** |  | **-** | **-** | **-** |
| UPTN | 46^a^ | 7 | 98^b,1^ |  | **-** | **-** | **-** |  | **-** | **-** | **-** |  | **-** | **-** | **-** |
| MJCN | **-** | **-** | **-** |  | 49^a^ | 4 | 79^c,2^ |  | 49^a^ | 4 | 125^b,1^ |  | 49^a^ | 4 | 126^b,1^ |
| MJTN | **-** | **-** | **-** |  | 48^a^ | 4 | 68^d,2^ |  | 51^a^ | 4 | 99^d,1^ |  | 47^a^ | 4 | 96^d,1^ |
| SACN | **-** | **-** | **-** |  | 49^a^ | 4 | 72^d,2^ |  | 49^a^ | 4 | 126^b,1^ |  | 49^a^ | 4 | 122^b,1^ |
| SATN | **-** | **-** | **-** |  | 49^a^ | 4 | 65^d,2^ |  | 51^a^ | 4 | 98^d,1^ |  | 51^a^ | 4 | 60^f,2^ |
| PBCN | **-** | **-** | **-** |  | 28^c^ | 5 | 99^b,3^ |  | 51^a^ | 4 | 126^b,1^ |  | 28^c^ | 4 | 121^b,2^ |
| PBTN | **-** | **-** | **-** |  | 30^c^ | 5 | 75^c,2^ |  | 51^a^ | 4 | 98^d,1^ |  | 52^a^ | 4 | 97^d,1^ |
| UPCP | 47^a^ | 5 | 105^b,1^ |  | **-** | **-** | **-** |  | **-** | **-** | **-** |  | **-** | **-** | **-** |
| UPTP | 47^a^ | 5 | 70^d,1^ |  | **-** | **-** | **-** |  | **-** | **-** | **-** |  | **-** | **-** | **-** |
| MJCP | **-** | **-** | **-** |  | 47^a^ | 3 | 68^d,2^ |  | 49^a^ | 3 | 105^c,1^ |  | 47^a^ | 4 | 101^c,1^ |
| MJTP | **-** | **-** | **-** |  | 47^a^ | 3 | 46^f,2^ |  | 49^a^ | 3 | 79^e,1^ |  | 47^a^ | 4 | 57^f,1^ |
| SACP | **-** | **-** | **-** |  | 46^a^ | 3 | 65^d,2^ |  | 48^a^ | 4 | 105^c,1^ |  | 45^b^ | 2 | 38^h,3^ |
| SATP | **-** | **-** | **-** |  | 47^a^ | 3 | 48^f,2^ |  | 48^a^ | 4 | 51^g,1^ |  | 47^a^ | 2 | 46^g,2^ |
| PBCP | **-** | **-** | **-** |  | 28^c^ | 3 | 61^e,1^ |  | 30^c^ | 2 | 44^h,3^ |  | 32^c^ | 4 | 49^g,2^ |
| PBTP | **-** | **-** | **-** |  | 37^b^ | 3 | 64^e,2^ |  | 40^b^ | 2 | 71^f,1^ |  | 44^b^ | 4 | 50^g,3^ |

^*^ UPCN= unprimed, control (unstressed), Nagina-22 UPCP= unprimed, control, Pusa Sugandh-5

UPTN= unprimed, treated (stressed), Nagina-22 UPTP= unprimed, treated, Pusa Sugandh-5

MJCN= MJ-primed, control, Nagina-22 MJCP= MJ-primed, control, Pusa Sugandh-5

MJTN= MJ-primed, treated, Nagina-22 MJTP= MJ-primed, treated, Pusa Sugandh-5

SACN= SA-primed, control, Nagina-22 SACP= SA-primed, control, Pusa Sugandh-5

SATN= SA-primed, treated, Nagina-22 SATP= SA-primed, treated, Pusa Sugandh-5

PBCN= PB-primed, control, Nagina-22 PBCP= PB-primed, control, Pusa Sugandh-5

PBTN= PB-primed, treated, Nagina-22; PBTP= PB-primed, treated, Pusa Sugandh-5.

^Ψ^ FB= shoot fresh biomass recorded 4 days after withholding irrigation for imposition of drought stress. The values are means of three replications. Means followed by different *lowercase letters* in a *column*, different *numbers* in a *row* for fresh biomass are significantly different (*P* ≤0.05) by DMRT.

**Supplementary Table S2.** Estimation of soil moisture content (SMC) in the pots for contrasting rice genotypes: Nagina-22 (drought-tolerant) and Pusa Sugandh-5 (drought-sensitive) on different days after withholding irrigation for imposition of drought stress.

| **Sample^*^** | **Soil moisture content (%)** | | | |
| --- | --- | --- | --- | --- |
|  | **0 day** | **2^nd^ day** | **4^th^ day** | **6^th^ day** |
| UPCN | 65.8 ±1.20^a^ | 65.8 ±1.21^a^ | 65.2 ±1.18^a^ | 64.6 ±1.17^a^ |
| UPTN | 60.4 ±1.09^a^ | 48.1 ±0.96^b^ | 35.0 ±0.67^c^ | 20.7 ±0.53^d^ |
| MJCN | 65.2 ±1.18^a^ | 63.0 ±1.17^a^ | 63.5 ±1.18^a^ | 62.5 ±1.16^b^ |
| MJTN | 52.5 ±1.02^a^ | 37.4 ±0.82^b^ | 25.8 ±0.61^c^ | 18.1 ±0.51^d^ |
| SACN | 60.4 ±1.14^a^ | 61.4 ±1.15^a^ | 59.4 ±1.12^a^ | 57.1 ±1.01^b^ |
| SATN | 61.9 ±1.14^a^ | 46.5 ±0.91^b^ | 29.4 ±0.75^c^ | 23.4 ±0.57^d^ |
| PBCN | 59.9 ±1.15^a^ | 60.9 ±1.16^a^ | 61.9 ±1.16^a^ | 57.1 ±1.14^b^ |
| PBTN | 65.2 ±1.19^a^ | 55.4 ±1.03^b^ | 31.8 ±0.85^c^ | 25.3 ±0.64^d^ |
| UPCP | 63.5 ±1.18^a^ | 64.6 ±1.19^a^ | 63.5 ±1.18^a^ | 61.9 ±1.17^a^ |
| UPTP | 60.4 ±1.16^a^ | 52.9 ±1.01^b^ | 35.9 ±0.91^c^ | 22.5 ±0.55^d^ |
| MJCP | 63.5 ±1.14^a^ | 60.9 ±1.15^a^ | 63.5 ±1.18^a^ | 62.5 ±1.17^a^ |
| MJTP | 59.9 ±1.13^a^ | 44.1 ±0.84^b^ | 29.8 ±0.56^c^ | 23.9 ±0.51^d^ |
| SACP | 54.9 ±1.03^a^ | 54.1 ±1.02^a^ | 54.9 ±1.03^a^ | 54.1 ±1.03^a^ |
| SATP | 57.1 ±1.05^a^ | 50.6 ±10.1^b^ | 33.9 ±0.78^c^ | 21.4 ±0.47^d^ |
| PBCP | 59.0 ±1.09^a^ | 61.4 ±1.18^a^ | 59.9 ±1.12^a^ | 58.0 ±1.08^b^ |
| PBTP | 57.1 ±1.04^a^ | 44.6 ±0.85 ^b^ | 29.4 ±0.54^c^ | 24.4 ±0.49^d^ |

**^*^** UPCN= unprimed, control (unstressed), Nagina-22 UPCP= unprimed, control, Pusa Sugandh-5

UPTN= unprimed, treated (stressed), Nagina-22 UPTP= unprimed, treated, Pusa Sugandh-5

MJCN= MJ-primed, control, Nagina-22 MJCP= MJ-primed, control, Pusa Sugandh-5

MJTN=MJ-primed, treated, Nagina-22 MJTP= MJ-primed, treated, Pusa Sugandh-5

SACN= SA-primed, control, Nagina-22 SACP= SA-primed, control, Pusa Sugandh-5

SATN= SA-primed, treated, Nagina-22 SATP= SA-primed, treated, Pusa Sugandh-5

PBCN= PB-primed, control, Nagina-22 PBCP= PB-primed, control, Pusa Sugandh-5

PBTN= PB-primed, treated, Nagina-22; PBTP: PB-primed, treated, Pusa Sugandh-5.

The values are means ±SD for three replications. Means followed by different *lowercase letters* in a *row* are significantly different (*P* ≤0.05) by DMRT.
